# A Multicenter, Randomized, Single-Blind Trial Evaluating a Multi-Porous Urethral Catheter with Continuous Local Ropivacaine Infusion for the Reduction of Postoperative Catheter-Related Bladder Discomfort

**DOI:** 10.3390/jcm14124215

**Published:** 2025-06-13

**Authors:** Sangmin Lee, Kwang Taek Kim, Tae Beom Kim, Kyung Jin Chung, Kookjin Huh, Hwanik Kim, Sang Hoon Song

**Affiliations:** 1 Department of Urology, Asan Medical Center, University of Ulsan College of Medicine, Seoul 05505, Republic of Korea; sangminlee92@naver.com; 2 Department of Urology, Gachon University Gil Medical Center, Gachon University School of Medicine, Incheon 21565, Republic of Korea; shinekkt@gmail.com (K.T.K.); tbkim@gachon.ac.kr (T.B.K.); kjchung@gilhospital.com (K.J.C.); 3 Department of Urology, Hallym University Sacred Heart Hospital, Hallym University College of Medicine, Anyang 14068, Republic of Korea; janosacs@hallym.or.kr

**Keywords:** anesthetics, local, pain, postoperative, randomized controlled trial, ropivacaine, urologic surgical procedures, urinary catheterization

## Abstract

**Background/Objectives:** Catheter-related bladder discomfort (CRBD) commonly occurs in patients undergoing urologic surgery and significantly affects patient comfort and recovery. We evaluated the efficacy and safety of continuous local ropivacaine infusion using a specialized multi-porous urethral catheter in reducing postoperative CRBD. **Methods**: This multicenter, prospective, randomized, single-blind trial enrolled 136 male patients undergoing short-term catheterization after urologic surgery. Participants were randomized into three groups—a control group receiving saline infusion, Group 1 receiving 0.5% ropivacaine at 1 mL/h, and Group 2 receiving 0.5% ropivacaine at 2 mL/h—for up to 48 h via a multi-porous urethral catheter. The primary outcome was the incidence of CRBD at 24 h postoperatively. Secondary outcomes included changes in urethral pain assessed by a visual analog scale (VAS), urinary symptom scores, complication rates, and patient-reported catheter inconvenience and reuse intention using Likert scales. **Results**: The incidence of CRBD was significantly lower in Group 1 (19.6%) and Group 2 (11.1%) compared to the control group (44.4%; *p* = 0.001), demonstrating a clear dose–response relationship. Changes in urethral pain scores (VAS) from baseline were significantly lower in the ropivacaine groups compared to the control (*p* = 0.023). Complication rates were similar among groups (control 13.3%, Group 1 6.5%, Group 2 15.6%; *p* = 0.378), although catheter leakage occurred more frequently in Group 2, without statistical significance (*p* = 0.122). **Conclusions**: Continuous local ropivacaine infusion using a multi-porous urethral catheter effectively reduces the incidence of postoperative CRBD without increasing side effects. This approach may improve patient comfort during perioperative catheter management.

## 1. Introduction

Indwelling urinary catheters are routinely used during and after urologic surgeries to ensure adequate bladder drainage and prevent urinary retention. However, their use frequently results in catheter-related bladder discomfort (CRBD), characterized by suprapubic pain, urinary urgency, and discomfort despite continuous drainage [1]. CRBD is reported in 47–90% of patients undergoing catheterization, significantly affecting postoperative recovery by delaying mobilization, prolonging hospitalization, and reducing patient satisfaction [2,3].

Various pharmacologic strategies, including α-2 adrenergic agonists, anti-inflammatory analgesics, and antimuscarinic agents, have been evaluated to attenuate CRBD [4,5,6]. Although these systemic therapies can reduce CRBD symptoms, their clinical utility is often limited by side effects such as sedation, dry mouth, blurred vision, cognitive impairment, and increased risk of acute urinary retention (AUR) [6,7].

Consequently, there is growing interest in local and regional analgesic modalities that can target the bladder and urethral nociceptive pathways while minimizing systemic drug exposure. Advances in biomedical device engineering have fostered the development of urinary catheters capable of continuously eluting local anesthetics directly into the bladder or urethra, maintaining therapeutic drug concentrations at the pain source [8,9]. Specialized indwelling catheters capable of infusing lidocaine or ropivacaine locally have shown promise in early studies, significantly mitigating CRBD [10,11]. Among available local anesthetics, ropivacaine is particularly well-suited for such continuous infusion due to its prolonged duration of action and favorable safety profile compared to bupivacaine [12].

A recent multicenter pilot trial demonstrated that a urethral catheter with continuous ropivacaine infusion could reduce postoperative CRBD and urethral pain compared to standard saline irrigation in patients undergoing urologic procedures [10]. However, that pilot study was limited by a small sample size and restricted patient selection, which highlights the need for broader validation. Building on these findings, this multicenter, prospective, randomized trial aims to further validate the efficacy and safety of continuous local ropivacaine infusion through a multi-porous urethral catheter, potentially establishing this method in clinical practice.

## 2. Materials and Methods

### 2.1. Study Design and Ethics Approval

This multicenter, prospective, randomized, single-blind clinical trial was conducted at three medical centers in South Korea (Asan Medical Center, Hallym University Sacred Heart Hospital, and Gachon University Gil Medical Center) from November 2023 to February 2025. The study protocol was approved by the Institutional Review Board of Asan Medical Center (IRB No. 2023-0968) and registered with the Clinical Research Information Service (KCT0008598). This investigation was designed as an extension of a previously published pilot study, with an expanded cohort and newly collected data [10]. All procedures adhered to the ethical principles of the Declaration of Helsinki, and written informed consent was obtained from each participant before enrollment.

### 2.2. Participants and Randomization

Eligible participants were male patients aged 19–80 years scheduled for urologic surgery requiring short-term (<7 days) urethral catheterization under general anesthesia. Inclusion criteria were an Eastern Cooperative Oncology Group (ECOG) performance status of 0 or 1, intact cognitive function, and the ability to provide informed consent. Exclusion criteria were prior pelvic surgery or radiation, preoperative or planned oncologic therapy, hypersensitivity to local anesthetics, severe hypertension, spinal anesthesia, anticipated catheterization exceeding 7 days, and refusal or unsuitability for trial participation.

After obtaining informed consent, baseline evaluations including urethral pain assessed by visual analog scale (VAS), International Prostate Symptom Score (IPSS), Overactive Bladder Symptom Score (OABSS), and Overactive Bladder Questionnaire-8 (OAB-V8) were performed before randomization. Patients were then randomized in a 1:1:1 ratio into three groups (control, Group 1, and Group 2) using centralized stratified block randomization based on preoperative urethral pain (VAS ≤ 2 vs. >2). Patients were blinded to their group assignment. Additionally, the statistical analysis investigator remained blinded to group allocation until the entire data collection process was completed.

### 2.3. Intervention and Procedures

A specialized multi-porous urethral catheter (FreeFoley™, S&S Med, Gunpo, Republic of Korea; 16 French (Fr)) was placed under sterile conditions following surgery. The FreeFoley™ system integrates a multi-perforated catheter shaft with an elastomeric pump (Neofuser™, S&S Med, Gunpo, Republic of Korea), enabling continuous local delivery of anesthetic directly along the urethral mucosa. Continuous infusion of the assigned solutions was initiated via an elastomeric pump immediately postoperatively: control group received saline at 1 mL/h, Group 1 received 0.5% ropivacaine at 1 mL/h (5 mg/h), and Group 2 received 0.5% ropivacaine at 2 mL/h (10 mg/h) for up to 48 h. The infusion rate was set in advance and passively maintained by the elastomeric pump’s internal pressure system, requiring no manual adjustment by clinical staff. Each patient received a total dose of up to 240 mg or 480 mg of ropivacaine, depending on the assigned group. Catheter setup and use were comparable to standard Foley catheter care and did not require additional training or staffing. The elastomeric pump was managed similarly to a postoperative patient-controlled analgesia (PCA) device and did not impose any additional care burden. Detailed technical descriptions of the device configuration and drug delivery mechanisms have been reported previously [10]. Foley catheters were maintained until at least the morning of postoperative day 1, after which removal timing was determined based on individual institutional surgical protocols.

### 2.4. Data Collection and Clinical Assessments

Baseline demographic data collected included age, comorbidities, previous urologic surgery, catheterization history, presence of benign prostatic hyperplasia (BPH), and current urologic medications such as alpha-blockers or antimuscarinics.

Clinical assessments, including urethral pain (VAS), IPSS, OABSS, and OAB-V8 scores, were conducted preoperatively before randomization. These evaluations were repeated at 24 h postoperatively to assess changes from baseline. The degree of inconvenience experienced during catheter use and willingness to reuse the catheter system were also evaluated 24 h postoperatively using validated 5-point Likert scales. All complications during the study period were documented and graded according to the Clavien–Dindo classification system [13]. The total number of doses administered for each systemic analgesic was recorded. This included nonsteroidal anti-inflammatory drugs (NSAIDs), tramadol, pethidine, and fentanyl. To compare systemic analgesic exposure across groups, the average number of doses per patient was calculated.

### 2.5. Outcomes

The primary outcome of this study was the incidence of CRBD at 24 h postoperatively. CRBD was defined as an increase of two or more points in the urethral pain (VAS) score compared to the preoperative baseline assessment.

Secondary outcomes included change in urethral pain score (VAS), complication rates such as catheter leakage and AUR, and patient-reported outcomes, including the degree of inconvenience experienced during catheter use and willingness to reuse the catheter system, assessed using validated 5-point Likert scales.

### 2.6. Statistical Analysis

Sample size calculation was based on a previous pilot study [10] that evaluated changes in urethral pain visual analog scale (VAS) scores following catheterization, which showed mean differences of approximately 0.6 to 0.7 points between groups. Based on these observed differences and assuming a conservative estimate of a medium-to-large effect size (f = 0.4), a priori power analysis was conducted using G*Power version 3.1.9.7 with α = 0.05, power = 0.95, and three parallel groups. The analysis indicated that a minimum of 102 participants would be required. Allowing for an anticipated 10% dropout rate, this study aimed to recruit at least 112 participants to ensure adequate statistical power.

Categorical variables were analyzed using chi-square tests or Fisher’s exact tests, as appropriate. Continuous variables were summarized as mean ± standard deviation and compared among groups using one-way ANOVA or Kruskal–Wallis tests, depending on data normality. Post hoc pairwise comparisons between groups were performed using Mann–Whitney U tests. To assess whether catheter leakage influenced patients’ willingness to reuse the catheter system, willingness to reuse scores were compared between patients with and without leakage using the Mann–Whitney U test. All statistical analyses were two-tailed, and a *p*-value of less than 0.05 was considered statistically significant. Statistical analyses were conducted using R software (version 4.3.2).

## 3. Results

A total of 137 patients were screened, and 136 completed the study and were included in the final analysis. One patient from Group 1 was excluded due to postoperative transfer to the intensive care unit for hypoxia. Baseline demographic and clinical characteristics were well-balanced across all three groups, with no statistically significant differences except for hypertension, which was slightly more prevalent in Group 2 (*p* = 0.050) (Table 1). Surgical procedures included, but were not limited to, ureteroscopic stone surgery, robot-assisted partial nephrectomy, radical nephrectomy, percutaneous nephrolithotomy, cystolitholapaxy, hydrocelectomy, and circumcision. Ureteroscopic stone surgery was the most frequently performed across all groups.

**Figure 1:**
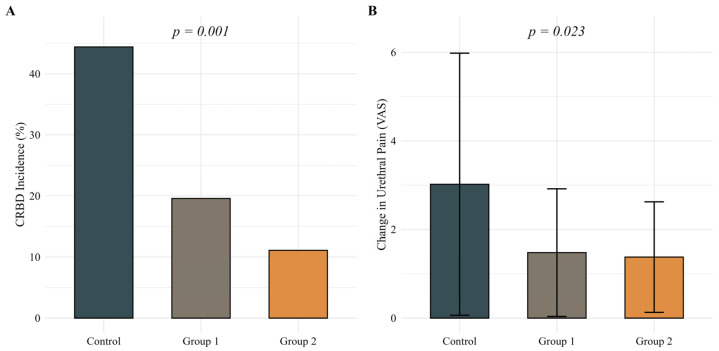
(**A**) Incidence of catheter-related bladder discomfort (CRBD) at 24 h postoperatively and (**B**) changes in urethral pain scores (VAS) from baseline to 24 h postoperatively across treatment groups. Statistical analysis was performed using the chi-square test for Panel A and the Kruskal–Wallis test for Panel B. A *p*-value < 0.05 was considered statistically significant.

The incidence of CRBD at 24 h postoperatively was significantly lower in Group 1 (19.6%) and Group 2 (11.1%) compared with the control group (44.4%; *p* = 0.001), demonstrating a clear dose–response relationship (Figure 1A). Changes in urethral pain scores (VAS) from baseline to 24 h postoperatively were significantly lower in both ropivacaine groups compared with the control group (mean ± SD: control 3.0 ± 3.0; Group 1 1.5 ± 1.4; Group 2 1.4 ± 1.2; *p* = 0.023) (Figure 1B).

Overall complication rates, including hematuria, dysuria, catheter leakage, and dysfunctional voiding, were comparable among groups (control 13.3%, Group 1 6.5%, Group 2 15.6%; *p* = 0.378). Specifically, catheter leakage occurred more frequently in Group 2 (13.3%) compared to the control group (6.7%) and Group 1 (2.2%), though the difference was not statistically significant (*p* = 0.122). Notably, no cases of AUR occurred in any group. All reported complications were mild and classified as Clavien–Dindo grade 1 (Table 2). The average number of doses per patient for each systemic analgesic, including NSAIDs, tramadol, pethidine, and fentanyl, was evaluated across groups. No statistically significant differences were observed (all *p* > 0.05). Detailed information is provided in Supplementary Table S1.

**Figure 2:**
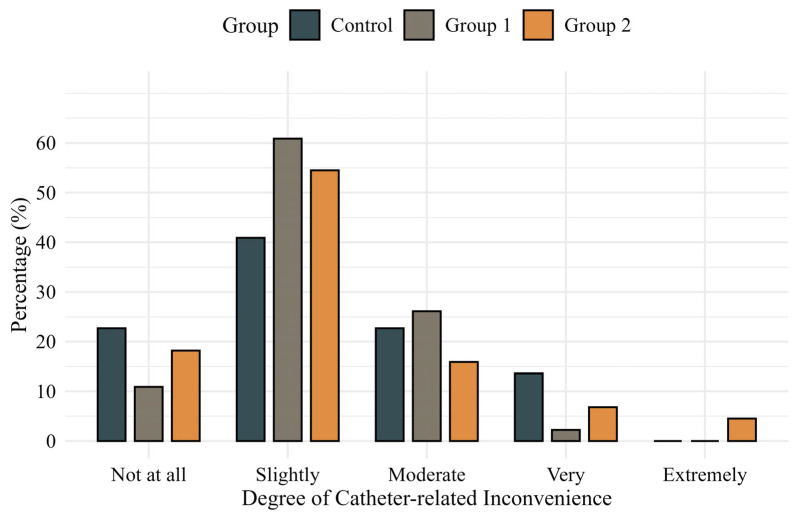
Patient-reported degree of inconvenience during catheter use measured by a validated 5-point Likert scale.

**Figure 3:**
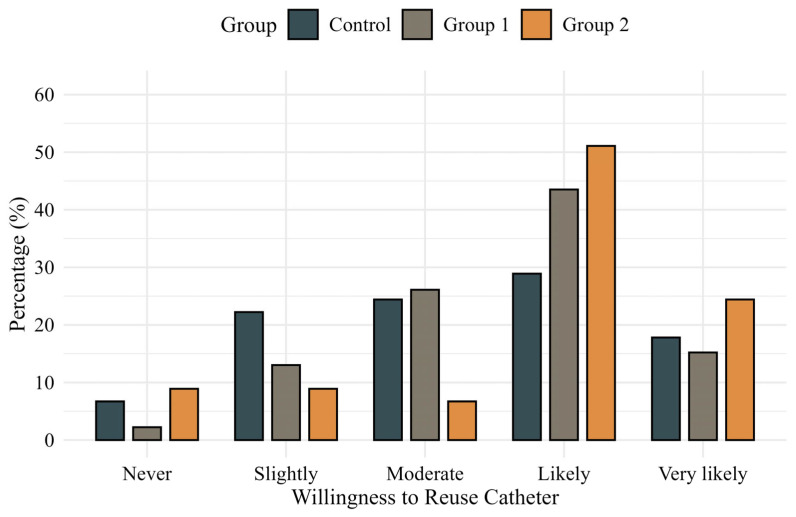
Patient-reported willingness to reuse the catheter system assessed using a validated 5-point Likert scale.

Comparisons of IPSS, International Prostate Symptom Score Quality of Life (IPSS-QOL), OABSS, and OAB-V8 scores at baseline and 24 h postoperatively showed no significant differences among the groups. Patient-reported inconvenience related to catheter use at 24 h postoperatively was also similar across groups (*p* = 0.558) (Figure 2). However, willingness to reuse the catheter system showed a favorable trend in Group 2, reaching borderline significance in a pairwise comparison with the control group (*p* = 0.050) (Figure 3). In additional analyses, patients who experienced catheter leakage reported a significantly lower willingness to reuse the catheter system than those who did not (*p* = 0.044).

## 4. Discussion

This multicenter, randomized, controlled trial demonstrated that continuous local ropivacaine infusion using a specialized multi-porous urethral catheter significantly reduced the incidence of postoperative CRBD in patients undergoing urologic surgery. Specifically, CRBD incidence at 24 h postoperatively was significantly lower in both ropivacaine infusion groups compared to the control group, which confirms the efficacy of continuous local anesthetic infusion in alleviating CRBD. These findings align with prior studies suggesting that targeted local analgesia can effectively decrease CRBD by directly anesthetizing the urethra and bladder mucosa.

CRBD is a common and distressing postoperative issue, reported in 47–90% of catheterized patients, depending on the diagnostic criteria [1]. If mild symptoms are included, over 80% of patients report discomfort, and 27–55% experience moderate-to-severe CRBD [14]. This variability underscores how differing definitions and assessment methods can impact reported incidence and outcomes. Our study defined CRBD strictly as an increase of two or more points on the urethral pain VAS from baseline, which indicates clinically significant discomfort. This definition likely explains the relatively lower incidence observed in our control group compared to those in other studies. Even with this conservative definition, our results clearly demonstrated that continuous ropivacaine infusion effectively reduced the incidence of CRBD, emphasizing the clinical relevance and efficacy of this local analgesic strategy.

Our results align with growing evidence supporting local anesthetic techniques for CRBD management, such as intravesical instillation of lidocaine or bupivacaine. In a randomized study, bladder irrigation with dilute lidocaine immediately after surgery reduced moderate-to-severe CRBD by nearly 80% in the first 2 h after surgery, though no significant difference was noted after 6 h [15]. Similarly, intravesical bupivacaine installation dramatically lowered early CRBD incidence (16.2% vs 90.3% in controls) during anesthesia recovery [16]. These reports corroborate the concept that anesthetizing the bladder locally can prevent the intense urgency and burning sensation provoked by the catheter. Unlike single instillation methods, which typically have a relatively short duration of action, our continuous infusion method provides ongoing delivery of ropivacaine, maintaining bladder analgesia throughout the indwelling period. Indeed, we observed a sustained reduction in CRBD up to 24 h postoperatively in the ropivacaine infusion group compared to controls.

Despite its effectiveness, our continuous infusion method encountered practical challenges such as catheter leakage, which was observed more frequently in the high-dose infusion group. Higher infusion rates may increase leakage, potentially reducing the effective intravesical anesthetic concentration and therapeutic efficacy. Notably, our additional analysis revealed that patients experiencing catheter leakage reported significantly lower willingness to reuse the catheter system compared to those without leakage. These leakage events may help explain why a subset of patients in the higher-dose group responded with ‘very’ or ‘extremely’ high levels of catheter-related inconvenience on the Likert scale, despite the overall analgesic benefit. This finding underscores that leakage negatively impacts patients’ overall satisfaction and perception of catheter usability, suggesting that leakage management is crucial for therapeutic efficacy, patient acceptance, and practical adoption. Thus, addressing leakage through improved catheter designs or refined infusion techniques is essential for optimizing drug delivery and enhancing patient comfort and adherence. Nonetheless, significant analgesic benefits were still achieved despite this limitation.

Beyond local anesthetic techniques, various systemic pharmacological strategies have been explored to manage CRBD. Antimuscarinic agents such as oxybutynin, tolterodine, and solifenacin have been extensively studied for their role in suppressing involuntary detrusor contractions. A recent meta-analysis of 11 trials found that perioperative antimuscarinics significantly reduce CRBD incidence within the initial six postoperative hours without significantly increasing anticholinergic side effects compared to placebo, suggesting good short-term tolerability [6]. While effective, antimuscarinics generally have a delayed onset and incomplete efficacy in severe cases. Moreover, they carry an increased risk of AUR, particularly in susceptible patient populations, with a significantly elevated risk, with a reported relative risk of 8.3 (95% CI 4.8–14.2) during the initial 30 days of treatment [17]. In our study, antimuscarinics were avoided to isolate the local effects of ropivacaine; however, combining these agents with local anesthetics might be beneficial in practice.

Systemic analgesics and sedatives such as dexmedetomidine, tramadol, and ketamine have demonstrated efficacy in reducing CRBD; however, these agents frequently cause sedation, cognitive impairment, and prolonged recovery periods, potentially complicating postoperative management [14]. For instance, dexmedetomidine effectively reduces both the incidence and severity of CRBD, but common adverse events such as hypotension and bradycardia have been reported in previous clinical trials [18]. Similarly, tramadol and low-dose ketamine can alleviate symptoms but may lead to sedation and other adverse effects [19,20].

Alternative pharmacologic approaches, including non-opioid analgesics such as nefopam and NSAIDs, gabapentinoids, and magnesium sulfate, have shown some promise; however, their effectiveness varies, and they may not be consistently available or sufficient in isolation [7,21,22]. Local and regional techniques, including peripheral nerve blocks, effectively reduce CRBD incidence but are invasive and provide only a limited duration of analgesia [23,24]. By contrast, continuous ropivacaine infusion via a specialized multi-porous urethral catheter offers sustained, targeted analgesia directly to the bladder mucosa, avoiding systemic sedation and the invasive nature of nerve blocks, thereby providing optimal patient comfort and safety throughout the catheterization period.

Compared to our previous pilot study involving the same catheter system [10], the present trial was designed with an expanded sample size and enhanced methodological rigor to validate the initial findings in a broader clinical context. While the pilot study demonstrated promising outcomes with 60 patients, the current multicenter study more than doubled the cohort, allowing for stratified randomization based on baseline urethral pain and improved statistical power. Furthermore, the definition of CRBD was standardized using a ≥2-point increase in VAS, allowing for a more reproducible assessment of symptomatic change. Although both studies incorporated patient-reported measures using Likert scales, this study extended the analysis by evaluating the association between catheter leakage and reuse intention, revealing a significant relationship (*p* = 0.044). These refinements not only confirmed the analgesic efficacy observed previously but also provided more profound insights into practical usability and patient acceptance.

Our study contributes to the existing literature by highlighting a practical approach to improving postoperative patient comfort in urologic care. Continuous ropivacaine infusion via a specialized multi-porous urethral catheter proved effective and safe in reducing CRBD, which supports its clinical utility. Effective CRBD management is clinically significant, as it can prevent catheter-related agitation, reduce the need for supplementary analgesics or sedatives, and facilitate early postoperative mobilization, thus enhancing overall patient recovery and satisfaction [1,3]. Our findings indicate that continuous intravesical anesthetic delivery can be an advantageous addition to CRBD management strategies, particularly beneficial for patients at risk or intolerant of systemic medications. Future research should aim to further validate these findings through larger-scale studies and direct comparisons with traditional systemic therapies. Additionally, exploring combined modalities, such as concurrent low-dose antimuscarinic administration alongside local anesthetic infusion, could further optimize patient outcomes.

Despite promising results, this study has several limitations. The single-blind design could introduce potential assessment bias despite blinded evaluations, and the exclusive enrollment of male patients restricts generalizability to female populations. Additionally, variability in baseline analgesic regimens across participating institutions could have influenced patient-reported outcomes, potentially affecting the consistency of results. Additionally, our follow-up duration was limited to 24 h, which restricted the assessment of the longer-term effectiveness and safety of ropivacaine infusion during extended catheterization periods. Further studies investigating prolonged catheterization periods are warranted. Moreover, this study employed only two infusion rates of ropivacaine, which limited the ability to identify optimal dosing regimens; future dose–response studies would be beneficial in establishing comprehensive guidelines for clinical practice.

## 5. Conclusions

In conclusion, continuous ropivacaine infusion via a specialized multi-porous urethral catheter significantly reduces CRBD and enhances patient comfort during postoperative catheterization without increasing systemic adverse effects or complications. This technique provides sustained, targeted analgesia, an advantage over systemic pharmacological and invasive regional methods. Further catheter technology refinement, investigation into optimal dosing strategies, and potential combinations with adjunctive therapies will be crucial for advancing comprehensive CRBD management and improving patient outcomes following urologic surgery.

## Figures and Tables

**Table 1:** Baseline Demographic and Clinical Characteristics by Treatment Group.

Characteristic	Control (n = 45)	Group 1 (n = 46)	Group 2 (n = 45)	*p*-Value
Age, years	56.7 ± 11.4	51.9 ± 13.6	56.5 ± 13.6	0.145
ASA Grade				0.492
1–2	38.0 (84.4%)	35.0 (76.1%)	38.0 (84.4%)	
3–4	7.0 (15.6%)	11.0 (23.9%)	7.0 (15.6%)	
Weight, kg	72.4 ± 10.8	73.2 ± 11.8	77.5 ± 15.7	0.262
BMI, kg/m^2^	25.0 ± 3.4	24.8 ± 3.8	26.6 ± 4.2	0.076
Diabetes Mellitus	11.0 (24.4%)	12.0 (26.1%)	17.0 (37.8%)	0.317
Hypertension	24.0 (53.3%)	17.0 (37.0%)	28.0 (62.2%)	0.050
History of Urologic Surgery	11.0 (24.4%)	15.0 (32.6%)	14.0 (31.1%)	0.662
Previous Catheter History	23.0 (51.1%)	21.0 (45.7%)	21.0 (46.7%)	0.858
Benign Prostatic Hyperplasia	7.0 (15.6%)	6.0 (13.0%)	5.0 (11.1%)	0.823
Current Urologic Medication	17.0 (37.8%)	13.0 (28.3%)	20.0 (44.4%)	0.274
Type of Surgery				0.978
Ureteroscopic stone surgery	29.0 (64.4%)	31.0 (67.4%)	32.0 (71.1%)	
Renal surgery	12.0 (26.7%)	11.0 (23.9%)	10.0 (22.2%)	
Scrotal surgery	3.0 (6.7%)	3.0 (6.5%)	2.0 (4.4%)	
Bladder surgery	1.0 (2.2%)	0.0 (0.0%)	1.0 (2.2%)	
Circumcision	0.0 (0.0%)	1.0 (2.2%)	0.0 (0.0%)	
Length of Stay, days	1.91 ± 1.55	2.26 ± 2.13	1.62 ± 1.39	0.196
Length of Postoperative Catheterization, days	1.40 ± 0.78	1.17 ± 0.38	1.24 ± 0.77	0.158

ASA, American Society of Anesthesiologists; BMI, Body Mass Index. Values are presented as mean ± standard deviation or number (percentage). Categorical variables were compared using the chi-square test or Fisher’s exact test, and continuous variables were analyzed using one-way ANOVA or the Kruskal–Wallis test, depending on data distribution. A *p*-value < 0.05 was considered statistically significant.

**Table 2:** Postoperative Clinical Outcomes and Patient-Reported Measures by Treatment Group.

	Control (n = 45)	Group 1 (n = 46)	Group 2 (n = 45)	*p*-Value
Preoperative Urethral Pain VAS	0.56 ± 1.27	0.46 ± 0.69	0.44 ± 0.66	0.990
Postoperative Urethral Pain VAS	3.58 ± 3.15	1.93 ± 1.50	1.82 ± 1.39	0.092
Overall Complication Rate	6 (13.3%)	3 (6.5%)	7 (15.6%)	0.378
Catheter Leakage	3 (6.7%)	1 (2.2%)	6 (13.3%)	0.122
Preoperative IPSS Total Score	8.1 ± 6.2	7.2 ± 5.1	9.1 ± 7.8	0.760
Postoperative IPSS Total Score	7.8 ± 6.0	6.6 ± 5.3	8.9 ± 7.4	0.428
Preoperative IPSS-QOL	2.3 ± 1.6	2.4 ± 1.3	2.4 ± 1.6	0.877
Postoperative IPSS-QOL	2.2 ± 1.5	2.2 ± 1.4	2.4 ± 1.5	0.895
Preoperative OABSS Total Score (n = 106)	3.3 ± 2.4	3.4 ± 2.9	3.3 ± 2.9	0.919
Postoperative OABSS Total Score	3.5 ± 2.5	3.3 ± 2.9	3.6 ± 3.0	0.796
Preoperative OAB-V8 Score (n = 30)	11.2 ± 3.4	10.9 ± 2.4	13.4 ± 7.4	0.823
Postoperative OAB-V8 Score	13.6 ± 7.2	10.7 ± 1.9	14.0 ± 9.2	0.799

IPSS, International Prostate Symptom Score; IPSS-QOL, International Prostate Symptom Score Quality of Life; OABSS, Overactive Bladder Symptom Score; OAB-V8, Overactive Bladder Questionnaire-8; VAS, Visual Analog Scale. Values are presented as mean ± standard deviation or number (percentage). Categorical variables were compared using the chi-square test or Fisher’s exact test, and continuous variables were analyzed using one-way ANOVA or the Kruskal–Wallis test, depending on data distribution. A *p*-value < 0.05 was considered statistically significant.

## Data Availability

The datasets generated and analyzed during the current study are available from the corresponding author on reasonable request.

## Supplementary Materials

The following supporting information can be downloaded at: https://www.mdpi.com/article/10.3390/jcm14124215/s1, Table S1: Mean number of doses per patient for each systemic analgesic by treatment group.

## Abbreviations

The following abbreviations are used in this manuscript:

CRBD	Catheter-related bladder discomfort
VAS	Visual Analog Scale
AUR	Acute Urinary Retention
IRB	Institutional Review Board
ECOG	Eastern Cooperative Oncology Group
IPSS	International Prostate Symptom Score
OABSS	Overactive Bladder Symptom Score
OAB-V8	Overactive Bladder Questionnaire-8
Fr	French
BPH	Benign Prostatic Hyperplasia
SD	Standard Deviation
ASA	American Society of Anesthesiologists
BMI	Body Mass Index
IPSS-QOL	International Prostate Symptom Score Quality of Life

## References

[1] Jang E.B., Hong S.H., Kim K.S., Park S.Y., Kim Y.T., Yoon Y.E., Moon H.S. (2020). Catheter-Related Bladder Discomfort: How Can We Manage It?. Int. Neurourol. J..

[2] Bai Y., Wang X., Li X., Pu C., Yuan H., Tang Y., Li J., Wei Q., Han P. (2015). Management of Catheter-Related Bladder Discomfort in Patients Who Underwent Elective Surgery. J. Endourol..

[3] Jeffery N., Mundy A. (2020). Innovations in indwelling urethral catheterisation. BJU Int..

[4] Zhang T., Li H., Lin C., An R., Lin W., Tan H., Cao L. (2024). Effects of an intraoperative intravenous Bolus Dose of Dexmedetomidine on postoperative catheter-related bladder discomfort in male patients undergoing transurethral resection of bladder tumors: A randomized, double-blind, controlled trial. Eur. J. Clin. Pharmacol..

[5] Shim J.W., Cha S., Moon H.W., Moon Y.E. (2022). Effects of Intraoperative Magnesium and Ketorolac on Catheter-Related Bladder Discomfort after Transurethral Bladder Tumor Resection: A Prospective Randomized Study. J. Clin. Med..

[6] Zhou Z., Cui Y., Zhang X., Lu Y., Chen Z., Zhang Y. (2021). The efficacy and safety of antimuscarinics for the prevention or treatment of catheter-related bladder discomfort: A systematic review and meta-analysis of randomized controlled trials. Perioper. Med..

[7] Bala I., Bharti N., Chaubey V.K., Mandal A.K. (2012). Efficacy of gabapentin for prevention of postoperative catheter-related bladder discomfort in patients undergoing transurethral resection of bladder tumor. Urology.

[8] Kallidonis P., Adamou C., Castillo S.V., Liourdi D., Liatsikos E., Lange D. (2021). Drug-delivering devices in the urinary tract: A systematic review. Arab. J. Urol..

[9] Kim C.R., Jang E.B., Hong S.H., Yoon Y.E., Huh B.K., Kim S.N., Kim M.J., Moon H.S., Choy Y.B. (2021). Indwelling urinary catheter assembled with lidocaine-loaded polymeric strand for local sustained alleviation of bladder discomfort. Bioeng. Transl. Med..

[10] Kim K.T., Shim M., Huh K., Song S.H., Uhm Y.J., Son I.T., Chung K.J., Kwak D.K., Choi Y.H., Kim H. (2024). Efficacy and Safety of Urethral Catheter with Continuous Infusion of Ropivacaine after Urologic Surgery: A Pilot Prospective Randomized Controlled Trial. J. Pers. Med..

[11] Imai H., Seino Y., Baba H. (2020). Efficacy of a novel urinary catheter for men with a local anesthetic injection port for catheter-related bladder discomfort: A randomized controlled study. J. Anesth..

[12] Zink W., Graf B.M. (2004). Benefit-risk assessment of ropivacaine in the management of postoperative pain. Drug Saf..

[13] Dindo D., Demartines N., Clavien P.A. (2004). Classification of surgical complications: A new proposal with evaluation in a cohort of 6336 patients and results of a survey. Ann. Surg..

[14] Lu J., Yang X., Zhang J., Huang Y. (2021). The efficacy of dexmedetomidine for the prevention of catheter-related bladder discomfort: A systematic review and meta-analysis. Medicine.

[15] Lin C.H., Lu I.C., Gau T.P., Cheng K.I., Chen H.L., Hu P.Y. (2024). Preventing Postoperative Catheter-Related Bladder Discomfort (CRBD) with Bladder Irrigation Using 0.05% Lidocaine Saline Solution: Monitoring with Analgesia Nociception Index (ANI) after Transurethral Surgery. Medicina.

[16] Pournajafian A., Ghodraty M.R., Shafighnia S., Rokhtabnak F., Khatibi A., Tavoosian S., Ghayoomi M. (2020). The Effect of Intravesical Diluted Bupivacaine on Catheter-Related Bladder Discomfort in Young and Middle-Aged Male Patients During Postanaesthetic Recovery. Turk. J. Anaesthesiol. Reanim..

[17] Martin-Merino E., Garcia-Rodriguez L.A., Masso-Gonzalez E.L., Roehrborn C.G. (2009). Do oral antimuscarinic drugs carry an increased risk of acute urinary retention?. J. Urol..

[18] Kim H.C., Hong W.P., Lim Y.J., Park H.P. (2016). The effect of sevoflurane versus desflurane on postoperative catheter-related bladder discomfort in patients undergoing transurethral excision of a bladder tumour: A randomized controlled trial. Can. J. Anaesth..

[19] Ozkan Sipahioglu F., Karaca Akaslan F., Yamankilic Mumcu O., Polat R., Sandikci F., Donmez A. (2022). Comparison of Different Strategies for Prevention of Catheter-Related Bladder Discomfort: A Randomized Controlled Trial. Med. Bull. Haseki.

[20] Safavi M., Honarmand A., Atari M., Chehrodi S., Amoushahi M. (2014). An evaluation of the efficacy of different doses of ketamine for treatment of catheter-related bladder discomfort in patients underwent urologic surgery: A prospective, randomized, placebo-controlled, double-blind study. Urol. Ann..

[21] Ren J., Yu T., Tian Y., Luo G. (2023). Comparative effectiveness of interventions for managing urological postoperative catheter-related bladder discomfort: A systematic review and network meta-analysis. BMC Urol..

[22] Jiang W., Zeng X., Zhou X., Liao O., Ju F., Zhao Z., Zhang X. (2023). Effect of magnesium sulfate perioperative infusion on postoperative catheter-related bladder discomfort in male patients undergoing laparoscopic radical resection of gastrointestinal cancer: A prospective, randomized and controlled study. BMC Anesthesiol..

[23] Li J.Y., Yi M.L., Liao R. (2016). Dorsal Penile Nerve Block With Ropivacaine-Reduced Postoperative Catheter-Related Bladder Discomfort in Male Patients After Emergence of General Anesthesia: A Prospective, Randomized, Controlled Study. Medicine.

[24] Bao X., Liu M., Li J., Yao H., Liu H., Tang G., Wang X., Zhou Z., Wu J., Cui Y. (2023). The efficacy of peripheral nerve block on postoperative catheter-related bladder discomfort in males: A systematic review and meta-analysis. Front. Surg..

