# Supplementary material for: A Multicenter, Randomized, Single-Blind Trial Evaluating a Multi-Porous Urethral Catheter with Continuous Local Ropivacaine Infusion for the Reduction of Postoperative Catheter-Related Bladder Discomfort
Source: J Clin Med. 2025 Jun 13;14(12):4215. doi: 10.3390/jcm14124215 (PMC12194525; doi:10.3390/jcm14124215)
Supplement: Supplementary file 1 [file jcm-14-04215-s001.zip › jcm-3667467-supplementary.pdf]

Supplementary Table S1. Mean number of doses per patient for each systemic analgesic by treatment group.

Systemic analgesic type	Control	Group 1	Group 2	p-value
**NSAIDs**	0.67 ± 0.88	0.78 ± 0.92	0.73 ± 0.94	0.835
**Tramadol**	0.78 ± 1.13	0.70 ± 1.03	0.56 ± 0.94	0.640
**Pethidine**	0.22 ± 0.47	0.28 ± 0.58	0.16 ± 0.52	0.267
**Fentanyl**	0.53 ± 0.76	0.54 ± 0.78	0.49 ± 0.76	0.918

* NSAIDs, Non-steroidal anti-inflammatory drugs

* Values are presented as mean ± standard deviation. Group comparisons were performed using the Kruskal–Wallis test. No statistically significant differences were observed among groups for any of the listed analgesics.
